# Human mesenchymal stromal cells from adipose tissue of the neck

**DOI:** 10.1007/s00405-012-1943-5

**Published:** 2012-02-05

**Authors:** Florian Böhrnsen, Nicole Rotter, Ulrich Lindner, Markus Meier, Barbara Wollenberg, Jürgen Rohwedel, Jan Kramer

**Affiliations:** 1 Institute of Virology and Cell Biology, University of Lübeck, Lübeck, Germany; 2 Medical Department I, University of Lübeck, Lübeck, Germany; 3 Present Address: Department of Oral and Maxillofacial Surgery, George-Augusta-University, Robert-Koch-Str. 40, 37075 Göttingen, Germany; 4 Department of Otolaryngology, Ulm University, Ulm, Germany; 5 Department of Otolaryngology, University of Lübeck, Lübeck, Germany

**Keywords:** Mesenchymal stem cells, Mesenchymal stromal cells, MSC, Tissue engineering, Tracheal transplantation, Adipose tissue, Tracheotomy

## Abstract

Mesenchymal stromal cells (MSC) have been introduced into the field of tissue-engineered airway transplantation. Since patients with extensive tracheal defects often require an open tracheotomy, this study investigated if MSC could be obtained from the adipose tissue of the neck during this procedure. Cells were isolated by plastic adherence from the adipose tissue of 8 patients. Cell isolates were analyzed for (i) proliferation, (ii) the expression of CD marker molecules and (iii) multilineage differentiation. The isolated spindle-shaped cells showed a high proliferation capacity and the flow cytometric analysis revealed a distinct population meeting the criteria for MSC. Using classical MSC cultivation protocols the characterized cells showed adipogenic, chondrogenic and osteogenic differentiation for all analyzed cell isolates. This study was able to demonstrate that sufficient amounts of stem/progenitor cells can be easily isolated from adipose tissue of the neck obtained during open tracheotomy. These cells may be a source for future tracheal replacement therapies.

## Introduction

Airway stenosis can lead to atelectasis and severe obstructive pneumonia. To secure airway function tracheotomy is often necessary. Moreover, tracheomalacia due to trauma, severe inflammation or malignant diseases may require extensive surgical treatment. Various therapeutic approaches for stenosis and obstruction of the trachea or bronchi have been reported, including bronchoscopic dilatation, laser ablation, airway stenting and surgical bronchoplasty [1, 2]. The current gold standard in surgical treatment of severe stenosis or defects is resection with end-to-end anastomosis. However, large airway defects (>5 cm) still present a major problem for clinicians because of the absence of effective methods of treatment. Recent studies have focused on the replacement of the cervical trachea or main bronchi based on the concept of in situ-tissue engineering [3, 4]. Attempts to provide synthetic grafts for application in humans have been associated with poor epithelization, exposure of the prosthesis matrix and poor functional outcome [5, 6]. Allograft transplants such as aorta, functioning as a biologic incubator, may allow host cells to migrate into the airway transplant in vivo [7]. In particular, after malignant or infectious diseases lifelong immunosuppressive medication is contraindicated, but it is necessary to avoid rejection of allogenic material. This restricts the clinical application of allografts. Therefore, autologous transplants are favored for replacement strategies. Autologous bone marrow mesenchymal stem cells have recently been introduced into the field of tissue-engineered airway transplantation. Besides autologous epithelial cells and chondrocytes, bone marrow-derived mesenchymal stem cells were used for the complete replacement of the left bronchus in a bioengineered human trachea [8]. Bone marrow mesenchymal stem cells have been found to differentiate along multiple mesenchymal lineages [9–11]. Even though it is commonly accepted that multipotent mesenchymal stem cells exist, there is clear evidence that isolation via plastic adherence results in heterogeneous populations including subsets of stem/progenitor cells with a contingently different stem cell potential. To point out this heterogeneity multipotent fibroblast colony-forming cells have been reclassified as mesenchymal stromal cells (MSC) [11]. Besides bone marrow, other sources of adult stem cells have been characterized, posting the hypothesis that stem cells capable of multilineage differentiation might reside in any post natal-organ [12]. Adipose-derived MSC possess a particular relevance for tissue engineering settings because these cells can be harvested and handled easily and effectively without donor site morbidity. In rats regeneration of the trachea using a bioengineered scaffold with adipose-derived MSC has already been reported [13].

This study investigated whether adult MSC can be retrieved during a tracheotomy from the adipose tissue of the neck. The cells were isolated from the adipose biopsies and characterized by their adherence to plastic culture dishes, their fibroblastoid morphology, their ability to proliferate in culture and by means of flow cytometric analysis. The stem/progenitor cell isolates showed characteristics of multipotent adult stem cells, and given the appropriate culture conditions, demonstrated multilineage differentiation into adipogenic, osteogenic and chondrogenic cells. Furthermore, there is strong evidence that these adipose tissue-derived cells meet the requirements for MSC [11] and may therefore be applicable for clinical tissue-engineered airway transplantation in the future.

## Materials and methods

### Isolation of human stem/progenitor cells

The isolation of human stem/progenitor cells from the adipose tissue surrounding the trachea was carried out with samples of eight patients, aged between 55 and 78 years, in accordance with the patients` informed consent (approval by the ethics committee, University of Lübeck, no. 05-218) and given indication for surgical tracheotomy. The biopsy was removed during the classic procedure of an open tracheotomy which was performed under general anesthesia in the operating room of the intensive care unit. Approximately 2–3 g of adipose tissue were removed from the neck as a routine procedure and immediately transferred to the laboratory for further processing. Adipose tissue samples were digested with basal medium supplemented with 1 mg/ml collagenase A (Sigma, München, Germany) for 45 min at 37°C, sieved, centrifuged, and washed with basal medium. The number of cells was determined and the cell suspension was plated onto 75 cm^2^ tissue culture flasks (TPP, Trasadingen, Switzerland). The number of cells obtained directly after isolation was around 4 × 10^5^ cells. Non adherent cells were removed by the first medium change after 2 days. Single colonies of adherent fibroblast-like cells were first visible after 72 h of cultivation. All cultivations were performed at 37°C and 5% CO_2_.

### Cultivation of human stem/progenitor cells

Stem/progenitor cells were cultured in basal medium consisting of Dulbecco’s modified Eagle’s medium (DMEM, Invitrogen, Paisley, U.K.) supplemented with 1% sodium pyruvate (PAA, Pasching, Austria), 1% l-glutamine (PAA, Pasching, Austria), 1% MEM non-essential amino acids (Invitrogen, Paisley, U.K.), 1% penicillin/streptomycin (PAA, Pasching, Austria) and 10% fetal bovine serum (PAA, Pasching, Austria). When adherent cells reached approximately 80–90% confluence, they were washed with phosphate-buffered saline (PBS), trypsinized and centrifuged for 5 min at 250*g*. The cells were plated at a density of 1 × 10^4^ cells/cm^2^ and passaged every 4–10 days. When reaching subconfluence, the cells were phenotyped using flow cytometric analysis (passage p4) or replated for differentiation. Passages used for differentiation in three independent samples per experimental group (*n* = 3) were p4, p6 and p8 in all stem/progenitor cell isolates. The daily doubling index was used to determine the proliferation and growth properties of the human stem/progenitor cells.

### Flowcytometric analysis of human stem/progenitor cells

Trypsin/EDTA-(0.25%) treated cells (4th passage) were washed twice with FACS buffer (PBS, 1% BSA and 0.1% NaN_3_), adjusted to approximately 5 × 10^5^ cells/ml and subsequently stained. A 100-μl cell suspension was incubated with either 10 μl phycoerythrin (PE)-conjugated monoclonal antibodies (mAbs) or 10 μl non-conjugated mAbs and a secondary goat anti-mouse IgG1-PE at 4°C for 30 min. To discriminate human stem/progenitor cells from cells of hematopoietic origin, isolates were stained for CD34 and CD45. In addition, the following antigens were included into the phenotyping profile: CD29, CD44, CD54, CD73, CD105, CD106, CD140b and CD166. Prior to the flow cytometric analysis, all samples were filled up to a total volume of 500 μl with FACS buffer. Cells were analyzed on a Cytomics FC 500 flow cytometer using cytomics CXP software (Beckman Coulter, Krefeld, Germany). At least 10,000 events were acquired and analyzed using a one-parametric protocol (FL2) and FSC/SSC dot plot diagram to exclude cell debris by gating. Non-specific isotype-matched controls were used to determine background fluorescence. All mAbs against the human antigens CD34, CD45, CD29, CD44, CD54, CD73, CD105, CD106, CD140b and CD166 were purchased from Becton–Dickinson (Heidelberg, Germany).

### Differentiation of human stem/progenitor cells

All differentiation assays have been carried out with three independent samples per experimental group (*n* = 3). Chondrogenic differentiation was performed using micro mass body (MMB) cultivation [14]. Cells were trypsinized, counted and basal medium was replaced by chondrogenic induction medium. Aliquots of 2 × 10^5^ cells in 0.5 ml chondrogenic induction medium were centrifuged at 65*g* in 15 ml polypropylene conical tubes. Chondrogenic induction medium consisted of basal medium supplemented with 0.1 μM dexamethasone (Merk, Darmstadt, Germany), 300 μM ascorbic acid (Sigma, München, Germany), 1 mM l-proline (Sigma, München, Germany), 10 ng/ml transforming growth factor (TGF) β_3_, (R&D, Wiesbaden, Germany) and 1% ITS premix (Becton–Dickinson, Heidelberg, Germany: 6.25 μg/ml insulin; 6.25 μg/ml transferrin; 6.25 μg/ml selenious acid; 1.25 mg/ml bovine serum albumin; 5.35 mg/ml linoleic acid). Samples of MMBs were taken for RNA-isolation (4 MMB per experimental time point per sample per day), histochemical or immunhistochemical analysis (1 MMB per sample per day) during the course of chondrogenic differentiation. MMBs prepared for histochemical and immunhisto-chemical staining were embedded in Tissue-Tek O.C.T. (Sakura Finetechnical, Tokyo, Japan), frozen at −80°C and cryosectioned (10 μm) for further analysis. To screen for proteoglycan deposits or marker protein expression within the chondrogenic MMBs, cryosections were fixed and stained with Alcian blue or immunostained. Uninduced MMBs were stained as negative controls.

To analyze adipogenic and osteogenic differentiation, isolated stem/progenitor cells were differentiated via monolayer protocols [10, 15]. Adipogenic and osteogenic induction of the stem/progenitor cells was performed at 80–90% confluence. To induce osteogenic differentiation cells were treated with osteogenic medium for 25 days. Osteogenic medium consisted of basal medium supplemented with 0.1 μM dexamethasone (Merk, Darmstadt, Germany), 10 mM β-glycerolphosphate (Sigma, München, Germany) and 300 μM ascorbic acid (Sigma, München, Germany). To induce adipogenic differentiation cells were treated with adipogenic induction medium and adipogenic maintenance medium for 25 days. Induction medium consisted of basal medium supplemented with 0.5 mM 3-isobutyl-1-methylxanthine (IBMX Sigma, München, Germany), 1 μM dexamethasone (Merk, Darmstadt, Germany), 200 μM indomethacin (Sigma, München, Germany) and 2 μM insulin (Sigma, München, Germany). Following a four-day induction period, the adipogenic induction medium was replaced with adipogenic maintenance medium consisting of basal medium supplemented with 2 μM insulin for 3 days. This cycle was repeated three times and ultimately followed by a four-day period of adipogenic maintenance culture.

Lipid accumulation during adipogenic differentiation was demonstrated by Sudan III staining. Cells were washed with PBS followed by staining with a 0.2% solution of Sudan III (Sigma, München, Germany) in 70% ethanol. Alkaline Phosphatase (AP) activity of stem/progenitor cells differentiating along the osteogenic lineage was demonstrated using the AP staining kit (Sigma, München, Germany).

### Quantitative analysis of histochemical staining

To analyze the differentiation of human adipose tissue-derived stem/progenitor cells from the neck by AP or Sudan III staining, ten areas of 0.77 mm^2^ for osteogenic differentiation and ten areas of 0.235 mm^2^ for adipogenic differentiation were quantified per sample per day. The stained areas were measured in relation to the total area of cells using ImageJ software (NIH, Bethesda, MD, USA) and quantified in percent. Chondrogenic differentiation was analyzed by measuring Alcian blue-positive stained areas in relation to the total area of the sectioned MMB using ImageJ software (NIH, Bethesda, MD, USA) and quantified in percent.

### Fluorescent immunostaining

Human stem/progenitor cells cultured on chamber slides or MMB cryosections were rinsed three times with PBS, fixed for 5 min with pre-cooled (−20°C) methanol-acetone at 4°C, washed four times with PBS and incubated at room temperature for 30 min with 7.5% bovine serum albumin. Specimens were then incubated for 1 h with a primary antibody in a humidified chamber at 37°C. Antibodies specific for the following proteins were used (designation, dilution ratio in PBS as well as references are given in parentheses): stromal cell surface marker (STRO-1; 1:50; [16]), collagen type II (II–II-6B3; 1:20; [17]), collagen type X (XAC9; 1:20; [18]), osteopontin (MPIIIB101; 1:20; [19]), bone sialoprotein I + II (WVID1(9C5); 1:20; [19]). The antibodies were obtained from the Developmental Studies Hybridoma Bank (University of Iowa, Iowa City, IA, USA). After rinsing four times with PBS, slides were incubated for 1 h at 37°C with either fluorescein isothiocyanate (FITC, Dianova, Hamburg, Germany; 1:200) or cyanine3 (Cy3, Dianova, Hamburg, Germany; 1:600) labeled anti-mouse IgG as well as 4′,6-Diamidino-2-phenylindole dihydrochloride (DAPI; Sigma, Taufkirchen, Germany). Slides were washed four times in PBS and briefly washed in distilled water. After immunostaining the specimens were embedded in Vectashield mounting medium (Vector, Burlingame, CA, USA) and analyzed with the fluorescence microscope Axioskop (ZEISS, Oberkochen, Germany). Negative controls were performed using the secondary antibody only.

### RT-PCR analysis

Stem/progenitor cells differentiated via monolayer or MMB were collected at different time points, washed twice with PBS and total RNA was isolated using a standardized RNA Isolation Kit (Macherey & Nagel, Düren, Germany). The RNA concentrations were determined by measuring the absorbance at 260 and 280 nm. Samples of 500 ng RNA were reverse transcribed using oligo-dT primer and Superscript II reverse transcriptase following the manufacturer’s recommendations (Invitrogen, Paisley, U.K.). Aliquots of 1 μl from the reverse transcriptase reactions were used for amplification of transcripts using primers specific for the analyzed genes and *Taq* polymerase according to the manufacturer’s instructions (Fermentas, St. Leon, Germany). Reverse transcriptase reactions were denatured for 2 min at 95°C, followed by amplification for 30–40 cycles of 40 s denaturation at 95°C, 40 s annealing at the primer-specific temperature and 50 s elongation at 72°C. Primers specific for the following genes were used (sequence, annealing temperature as well as size are given in parentheses): PPARγ (5′- AAA CTC TGG GAG ATT CTC CT -3′, 5′- TCT TGT GAA TGG AAT GTC TT -3′, 56°C, 247 bp) [20], aP2 (5′- GCT TTG CCA CCA GGA AAG TG -3′, 5′- ATG ACG CAT TCC ACC ACC AG -3′, 60°C, 279 bp) [20], C/EBPalpha (5′- AGA AAG GGG TGG AAA CAT AGG -3′, 5′- GAA AGC TGA GGG CAA AGG -3′, 58°C, 685 bp) [20], osteopontin (5′- ACT GAT TTT CCC ACG GAC CT -3′, 5′- CAT TCA ACT CCT CGC TTT CC -3′, 58°C, 199 bp), osteocalcin (5′- CTC ACA CTC CTC GCC CTA TT -3′, 5′- CGC CTG GGT CTC TTC ACT AC -3′, 58°C, 143 bp), collagen type II (5′- AGG CTC CCA GAA CAT CAC CT -3′, 5′- ACA GTC TTG CCC CAC TTA CC -3′ 55°C, 472 bp) [21], Aggrecan (5′- GCA GAG ACG CAT CTA GAA ATT G -3′, 5′- GGT AAT TGC AGG GAA CAT CAT T -3′, 55°C, 504 bp) [21] and GAPDH (5′- CCG CAT CTT CTT TTG CGT CGC -3′, 5′- GCA ACT GTG AGG AGG GGA GAT TCA G -3′, 55°C, 1100 bp). Electrophoretic separation of PCR products was carried out on 2% agarose gels [2% (w/v) agarose (Roth, Karlsruhe, Germany)], 0.7 ng/ml ethidium bromide (Roth, Karlsruhe, Germany). The fragments were analyzed by computer-assisted densitometry in relation to GAPDH gene expression. The densitometric values of each marker were calculated in relation to GAPDH. From these values the highest of each marker was taken as 100%. Distilled water and no-RT reactions were always included as a negative control.

### Statistical analysis

Statistical analysis was performed using SigmaPlot 2000 software (Systat, Erkrath, Germany) and calculated according to the student’s *t* test. Samples were analyzed at least in three independent experiments (*n* = 3).

## Results

### Successful establishment of stem/progenitor cells after tracheotomy

**Fig. 1:**
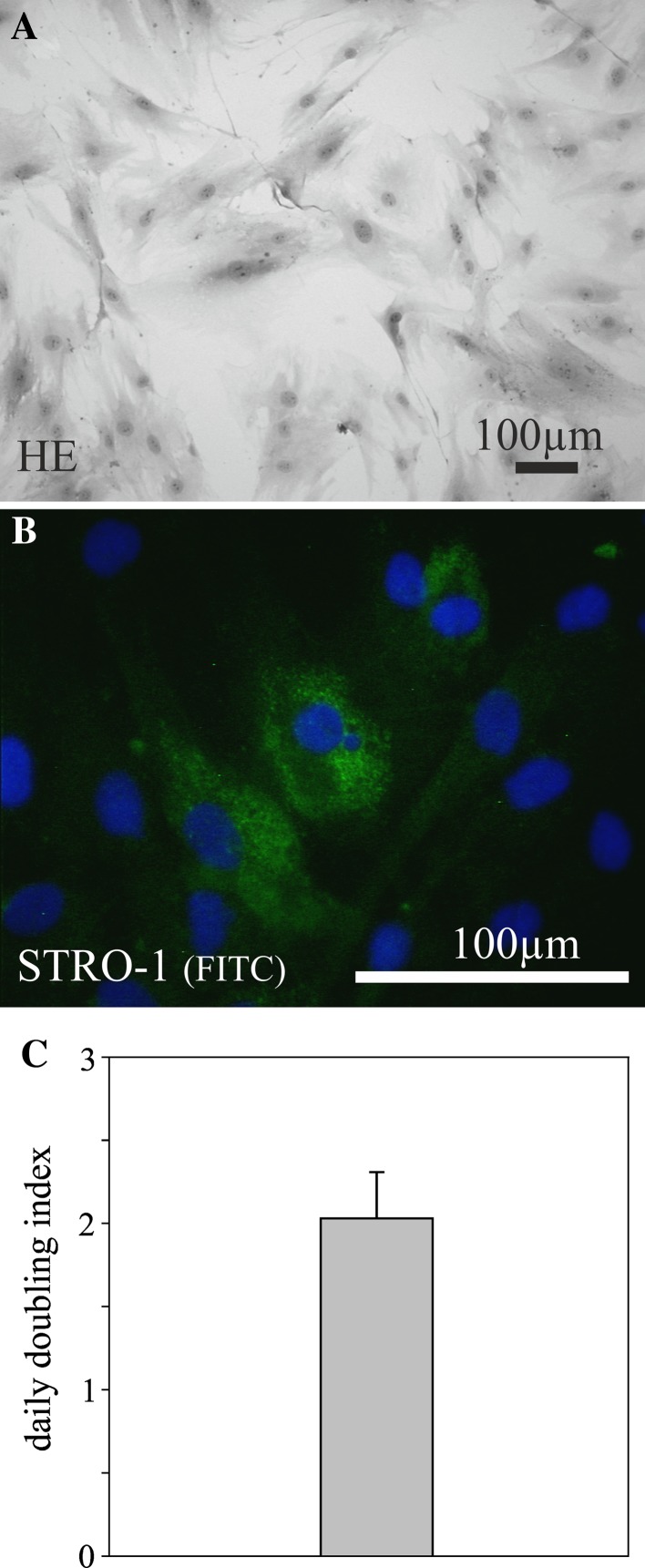
Hematoxylin-eosin (HE) staining of proliferating adipose tissue-derived cells from the neck (passage 4) isolated by plastic adherence after tracheotomy demonstrates a spindle-shaped morphology (**a**). The cells stained positive for STRO-1 (**b**) and showed a stable daily doubling index of 2.02 during further passages (**c**). Nuclei are stained *blue* using DAPI

During an open tracheotomy stem/progenitor cells were isolated from subcutaneous adipose tissue of eight patients, aged between 55 and 78 years. Plastic adherent cell populations from adipose tissue of the neck exhibited a typical spindle-shaped morphology (Fig. 1a). The first colonies of fibroblast-like cells could be observed after 72 h, with a 100% successful rate of stem/progenitor cell isolation. All cell isolates displayed stable growth characteristics, and STRO-1 could be detected in all isolates (Fig. 1b). Optimizing cell culture, an optimal cell growth and maintenance was achieved using a plating density of 1 × 10^4^ cells per cm^2^. During further passages, a daily doubling index of 2.02 was determined (Fig. 1c). The cells did not differentiate spontaneously during culture expansion into any morphologically identifiable cell type.

### Immunophenotypic characterization of stem/progenitor cells isolated from tracheotomy

**Fig. 2:**
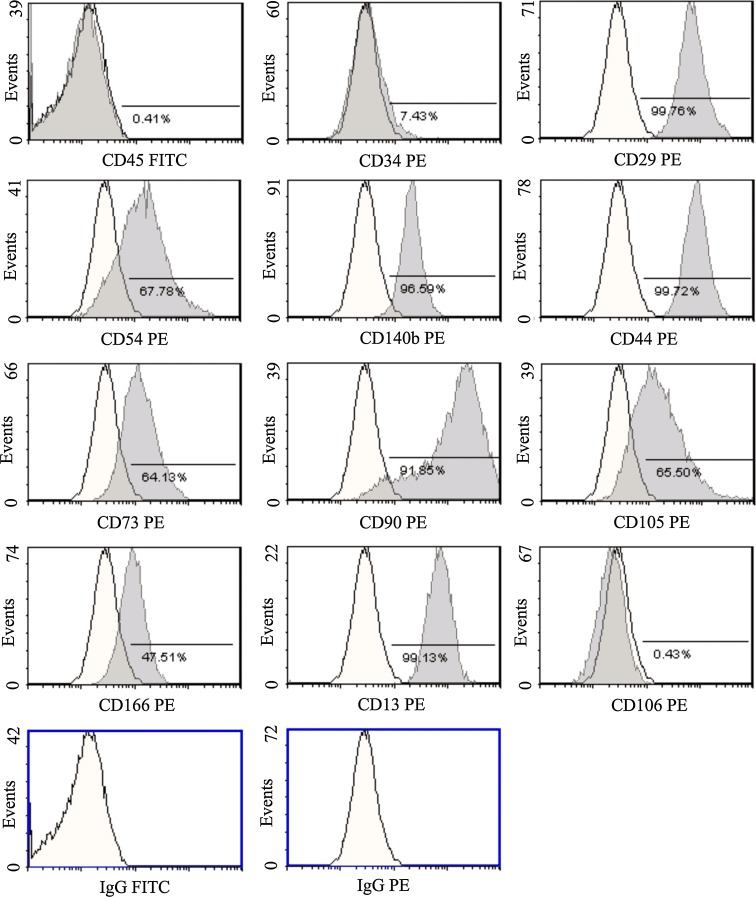
A representative flow cytometric analysis of adipose tissue-derived cells from the neck at the 4th passage is shown. Isotype-matched human antibody control staining is depicted in *white*. The specific markers are shown in *gray*

To characterize the isolated stem/progenitor cell population, CD surface antigen marker expression was analyzed by means of flow cytometric measurement (Fig. 2). The isolated cells showed a distinct phenotypic population (>90% homogeneous in passage 4). The stem/progenitor cell isolates were negative for CD45 (leukocyte common antigen) and CD34 (gp105-120), indicating that they were not of hematopoietic origin. The cells expressed CD29 (*b*1 integrin), CD54 (intercellular adhesion molecule-1) and CD140b (*b* platelet-derived growth factor receptor). Analysis for the hyaluronate receptor (CD44), ecto-5′-nucleotidase (CD73) and CD90 (thymocyte differentiation antigen-1, Thy-1) revealed strong expression. The matrix receptor CD105 (endoglin, SH2), the adhesion molecule CD166 (activated leukocyte cell adhesion molecule) and CD13 (aminopeptidase-N) were found to be expressed. The cells did not display an expression of CD106 (vascular cell adhesion molecule-1).

### Multilineage differentiation of tracheotomy-derived stem/progenitor cells

During multilineage differentiation and characterization of adipose tissue-derived cell cultures, the isolates were regularly analyzed by histochemical and immunofluorescence staining for adipogenic, osteogenic and chondrogenic cell types. RT-PCR analysis confirmed the multilineage differentiation of the isolated human stem/progenitor cells. We found that the amount of differentiated cells increased up to almost 70–90% after 25 days of cultivation in lineage-specific induction medium.

#### Adipogenic differentiation

**Fig. 3:**
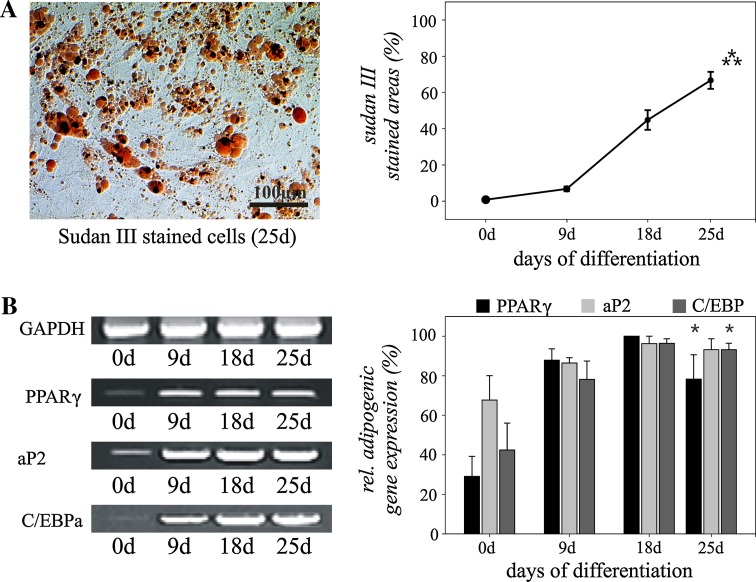
Adipogenic differentiation of human adipose-tissue derived stem/progenitor cells from the neck during monolayer cultivation. Sudan III staining demonstrates adipogenic differentiation with a maximum on day 25 after induction of differentiation (**a**). Analysis of relative marker gene expression confirmed adipogenic differentiation of the stem/progenitor cells (**b**). Mean values ± SEM derived from three independent experiments (*n* = 3) are shown. *Bar* = 100 μm. *p ≤ 0.05, ***p ≤ 0.001

To analyze the adipogenic differentiation, the stem/progenitor cells were induced in the expanded monolayer cultures by treatment with IBMX, dexamethasone, insulin and indomethacin. Morphologic changes as well as the formation of neutral lipid vacuoles were apparent as early as 1 week after induction and visualized by staining with Sudan III (Fig. 3a). Sudan III positivity was only observed after application of adipogenic medium. Initially, no staining was detectable in the stem/progenitor cell cultures. After 25 days of adipogenic induction, almost 70% of all cells were positive for the lipid staining and eventually filled with lipid vacuoles (Fig. 3a; 0 day vs. 25 days: *p* ≤ 0.001).

After 1 week, adipogenic induction of tracheotomy-derived stem/progenitor cells resulted in lineage-specific gene expression and increase of marker molecules such as the nuclear receptor *peroxisome proliferation*-*activated receptor γ* (*PPARγ*), the *fatty acid*-*binding protein* (*FABP*) *aP2* and the *CCAAT enhancer*-*binding protein alpha* (*C/EBPalpha*)—molecules controlling adipocyte differentiation and function (Fig. 3b; 0 day vs. 25 days: *p* ≤ 0.05).

#### Osteogenic differentiation

**Fig. 4:**
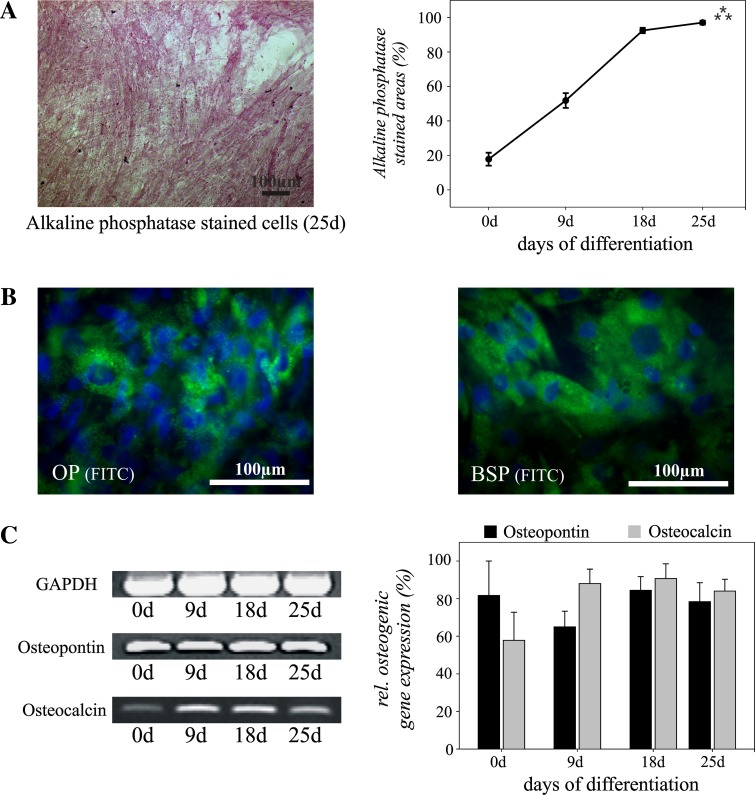
Osteogenic differentiation of human adipose-tissue derived stem/progenitor cells from the neck during monolayer cultivation. Alkaline phosphatase (AP) staining demonstrates osteogenic differentiation with a maximum on day 25 after induction of differentiation (**a**). Expression of osteopontin (OP) and bone sialoprotein (BSP) is shown by immunostaining. Nuclei are stained *blue* using DAPI (**b**). Analysis of relative marker gene expression confirmed osteogenic differentiation of the stem/progenitor cells (**c**). Mean values ± SEM derived from three independent experiments (*n* = 3) are shown. *Bar* = 100 μm. ***p ≤ 0.001

To assess the osteogenic differentiation, the stem/progenitor cells were induced by dexamethasone, glycerol phosphate and ascorbic acid in the presence of 10% FCS. As early as 1 week after induction, aggregates of cells showed expression of alkaline phosphatase (AP) which increased during the ongoing differentiation, resulting in almost 100% AP-positive cells on day 25 (Fig. 4b; 0 day vs. 25 days: *p* ≤ 0.001). Immunostaining revealed the expression of the osteogenic marker molecules osteopontin and bone sialoprotein (Fig. 4b). Both molecules could be detected after 9 days of osteogenic differentiation and the number of positive cells increased during further cultivation, indicating osteogenic differentiation. Before application of the osteogenic medium, no protein expression of bone sialoprotein was observed in the undifferentiated cell cultures by immunostaining, whereas a basal expression of osteopontin could be detected (data not shown).

These results were confirmed at the gene expression level by RT-PCR analysis (Fig. 4c). *Osteopontin* was already expressed in undifferentiated cell cultures, as it has been previously demonstrated for MSC [22]. However, *osteocalcin* demonstrated an increasing expression after 9 days of osteogenic differentiation and continued to be expressed at a high level until the end of the cultivation period, indicating formation of osteoblasts.

#### Chondrogenic differentiation

**Fig. 5:**
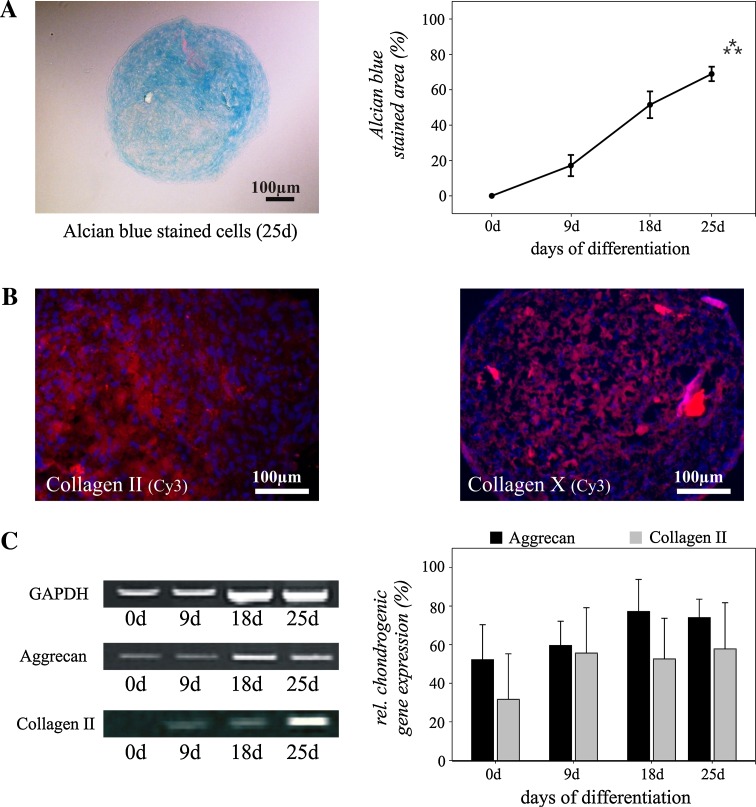
Chondrogenic differentiation of human adipose tissue-derived stem/progenitor cells from the neck. Alcian blue staining of cryosectioned “micromass bodies” (MMB) demonstrates chondrogenic differentiation with a maximum on day 25 after induction of differentiation (**a**). Expression of collagen type II and X is demonstrated by immunostaining. Nuclei are stained *blue* using DAPI (**b**). Analysis of relative marker gene expression confirmed chondrogenic differentiation of the stem/progenitor cells (**c**). Mean values ± SEM derived from three independent experiments (*n* = 3) are shown. *Bar* = 100 μm. ***p ≤ 0.001

To promote chondrogenic differentiation, we gently centrifuged the isolated cells to form a pelleted micromass, and cultured the micromass bodies (MMB) in a medium containing TGF-β_3_. Differentiation of tracheotomy-derived stem/progenitor cells along the chondrogenic lineage, using the classical MMB protocol, was characterized by Alcian blue-staining (Figure 5 a; 0 day vs. 25 days: *p* ≤ 0.001). All MMBs cultured in chondrogenic medium stained positive for Alcian blue after 18 days of cultivation demonstrating a proteoglycan-rich extracellular matrix. Immunostaining revealed the expression of collagen type II, the major component of hyaline cartilage (Fig. 5b). Collagen Type X, which is expressed by hypertrophic chondrocytes, was detected at the end of the 25-day differentiation period. Before application of chondrogenic medium, no protein expression of the analyzed chondrogenic marker molecules could be found in the undifferentiated cell cultures by immunostaining (data not shown).

RT-PCR analysis confirmed chondrogenic differentiation and expression of *collagen type II* and demonstrated the expression of *aggrecan*, the major proteoglycan of cartilage tissue (Fig. 5c). Taken together, these results demonstrate that all analyzed tracheotomy-derived stem/progenitor cells were able to differentiate along the chondrogenic lineage.

## Discussion

Cells used in tissue engineering can be derived from numerous sources, including primary tissues, cell lines and stem cells. Stem/progenitor cells contribute to the homeostasis and regeneration of tissue after trauma and disease [23, 24]. Many sources of adult stem cells have been characterized, posting the hypothesis that stem cells capable of multilineage differentiation might reside in any post natal-organ [12]. Among them, bone marrow MSC have been found to differentiate along multiple mesenchymal lineages [9–11] and have currently been used for the clinical transplantation of a tissue-engineered airway [8, 25]. However, it is necessary to obtain these cells by general anesthetic procedures, bone marrow biopsies or even operations. These methods may complicate the routine clinical scenario.

Ideally, cells used for clinical transplantation should be (1) easy to harvest, (2) highly proliferative, (3) autologous and non-immunogenic and (4) able to differentiate into a variety of cell types with specialized functions [26]. Most patients undergoing extensive airway or head and neck surgery require open tracheotomy to secure airway passage until recovery of swallowing and respiratory function is reached. In this study we investigated if stem/progenitor cells applicable for tissue engineering of the airway function could be easily isolated from adipose tissue of the neck during an open tracheotomy.

Characteristics that define mesenchymal stem/progenitor cells include high proliferative potential, ability to generate primary colony-forming unit-fibroblast and the ability to differentiate into bone, fat and cartilage. The multilineage potential of adult stem cells, however, has often been considered an attribute of heterogenic cell isolates [27, 28]. Therefore, minimal criteria have been denominated to define human MSC [11]. First, the cells are plastic adherent and second, they express the CD marker molecules CD105 (endoglin), CD73 (5′-nucleotidase) as well as CD90 (Thy1) and do not express the leukocyte marker molecule CD45 and the marker molecule CD34 for primitive hematopoietic progenitor cells. However, current studies demonstrated that MSC cannot be distinguished from fibroblasts by flow cytometric analysis using a panel of common marker molecules [29]. Furthermore, various markers that are used to purify MSC from marrow aspirates are rapidly down regulated following ex vivo expansion [30] or have been attributed to different subpopulations [31]. Therefore, the third criterion plays a pivotal role: MSC must show the multilineage capacity to differentiate into adipogenic, osteogenic and chondrogenic cells.

In this study we were able to demonstrate that proliferating cells could be easily isolated from adipose tissue surrounding the trachea by adherence to plastic culture dishes. The isolated cells showed a spindle-shaped morphology and expressed CD marker molecules characteristic for MSC [11, 30] and adipose tissue-derived stem cells [32]. Moreover, these cells were capable of differentiating efficiently into adipogenic, osteogenic and chondrogenic cell types under specific culture conditions in vitro. In addition, it has been shown that multipotential adipose tissue-derived stem cells accelerated neovascularization and epithelialization in an animal model of a tissue-engineered trachea [13]. Recently, there has also been evidence that fibroblasts, closely related to MSC populations [33, 34] enhanced epithelial differentiation and proliferation in an applied tracheal prosthesis [35].

## Conclusion

This study was able to verify the hypothesis that stem/progenitor cells can be easily isolated from adipose tissue of the neck during tracheotomy. The stem/progenitor cell isolates showed characteristics of multipotent adult stem cells, similar to those of bone marrow-derived MSC, and given the appropriate culture conditions demonstrated multilineage differentiation into adipogenic, osteogenic and chondrogenic cells. Furthermore, there is strong evidence that these adipose tissue-derived stem cells from the neck meet the requirements for MSC [11]. Thus, additional operative interventions to obtain bone marrow MSC through multiple time-intensive general anesthetic procedures, biopsies and operations may no longer be necessary to receive MSC-derived tissue-engineered airway transplantation. Direct sources of autologous, highly proliferative, multipotent stem cells for clinical transplantation which can be safely extracted during a one-time procedure will be pivotal for regenerative medicine in the future. To safely apply stem/progenitor cells in future medicine it will also be necessary to understand the integration and interaction of these cells with surrounding cellular structures [36]. The goal of future studies is to optimize the differentiation and application of adipose tissue-derived stem/progenitor cells from the neck for the reconstructive surgery of tracheal or bronchial airway.

### **Ethical conduct of research**

The authors state that they have obtained appropriate institutional review board approval or have followed the principles outlined in the Declaration of Helsinki for all human experimental investigations. In addition, for investigations involving human subjects, informed consent has been obtained from the participants involved.
